# Perioperative Intravenous Patient-Controlled Analgesic Efficacy of Morphine with Combined Nefopam and Parecoxib versus Parecoxib in Gynecologic Surgery: A Randomized, Double-Blind Study

**DOI:** 10.1155/2021/5461890

**Published:** 2021-02-12

**Authors:** Varinee Lekprasert, Lapuskorn Yapanan, Wichai Ittichaikulthol, Rungrawan Buachai, Phimol Soisod, Areepan Sophonsritsuk

**Affiliations:** ^1^Department of Anesthesiology, Faculty of Medicine Ramathidodi Hospital, Mahidol University, Bangkok 10400, Thailand; ^2^Reproductive Endocrinology and Infertility Unit, Department of Obstetrics and Gynaecology, Faculty of Medicine Ramathidodi Hospital, Mahidol University, Bangkok 10400, Thailand

## Abstract

**Background:**

Nefopam is a non-NSAIDs and opioid sparing centrally acting drug which is effective for a multimodal postoperative analgesia. The present study aimed to evaluate the analgesic efficacy of nefopam combined with parecoxib for gynecologic surgery.

**Methods:**

This randomized double-blinded control trial recruited participants (*n* = 72) who underwent gynecologic surgeries and divided them into either a nefopam or control group. The study group received parecoxib 40 mg plus nefopam 20 mg, while the control group received parecoxib 40 mg plus normal saline solution intravenously during open abdominal gynecological surgery. Both groups then received either nefopam or normal saline every 6 hours postoperatively for 24 hours. Intravenous patient-controlled analgesia with morphine was given for breakthrough pain within 24 h. The participants were evaluated for morphine consumption within 24 hours and postoperative pain using a verbal numerical rating scale (VNRS) at a postanesthetic care unit, at 6-, 12-, and 24-hour postoperative periods. Adverse effects were recorded.

**Results:**

Morphine consumption within 24 hours and adverse effects were not significantly different between both groups. Mean difference and 95% confident interval of morphine consumption between both groups was 1.00 (−4.56, 4.76), *P*=0.97. The VNRS on movement at 6 hours after surgery of the nefopam group was significantly different from that of the control group [mean (SD), 4.14 (2.11) vs. 5.14 (1.80), *P*=0.04]. The VNRS of the nefopam group at 12 hours after operation during resting and on movement was significantly different from that of the control group ([mean (SD), 1.47 (1.80) vs. 2.54 (2.15), *P*=0.03], [mean (SD), 3.22 (1.84) vs 4.17 (1.74), *P*=0.03]), respectively.

**Conclusions:**

The combined administration of nefopam and parecoxib during gynecologic surgery slightly reduced the VNRS at 6 and 12 hours postoperatively more than treatment with parecoxib.

## 1. Introduction

Postoperative pain is the most distressed adverse event occurred after major surgery which increases morbidity and prolongs the recovery period [1–3]. Multimodal analgesia based on a combination of different pharmacologic analgesic drugs alleviates this suffering pain [3, 4]. The advantages of this strategy include improving analgesia while decreasing doses of opioids and also reducing severity of adverse effects [3]. Drug used in multimodal analgesia including various groups of medication such as paracetamol, nonsteroidal inflammatory drugs (NSAIDs), and opioids when combined with nonopioids are not efficient in relieving pain [5].

Nefopam, a benzoxazocine derivative, was developed in the early 1970s as an antidepressant medication [6]. It is a non-NSAID and nonopioid analgesic (NOA) centrally acting drug which is used as a multimodal analgesia in the fields of general surgery, orthopedics, and gynecology [1, 2, 7]. The drug mechanism is mainly the inhibition of reuptake for serotonin, norepinephrine, and dopamine. Nefopam also modulates glutamatergic transmission by inhibiting N-methyl-D-aspartate (NMDA) receptors [8]. It exerted the synergistic action with ketoprofen in relieving moderate to severe pain after minor surgery [9] In addition, it decreased opioid consumption when used as a coanalgesic agent following laparoscopic abdominal surgery [8, 10]; however, no systemic NSAIDs were used in these study. The only study that demonstrated its role as a multimodal analgesia with systematic NSAIDs was performed in a patient undergoing total hip arthroplasty [11].

Previous studies comparing more than one NOA combined with morphine for postoperative surgery are very limited, with the majority of the studies being on opioid and one NOA, either NSAIDs, paracetamol, or nefopam, which showed the morphine sparing effect. Therefore, we hypothesized that the combination of 2 NOAs and opioid would produce an additional effect for decreasing postoperative pain after major abdominal surgery. The aim of this study was to investigate the analgesic efficacy and side effects of nefopam combined with parecoxib in open gynecologic surgery.

## 2. Materials and Methods

This prospective randomized controlled trial (RCT) was approved by the Ethical Clearance Committee on Human Rights Related to Researches Involving Human Subjects, Faculty of Medicine, Ramathibodi Hospital (MURA208/327).The study recruited patients who underwent gynecologic surgeries between August 2018 and July 2019. The participants and assessors were blinded to the treatment groups. This trial was registered at clinicaltrials.in.th (TCTR 20180808001).

All seventy-two patients aged 20–65 years with American Society for Anesthesiologists (ASA) physical status 1–2 who were scheduled for open hysterectomy under general anesthesia were enrolled to the study. The exclusion criteria included patient refusal to participate in the study, a body mass index (BMI) > 35 kg/m^2^, underlying diseases, i.e., liver failure (clinical and abnormal laboratory test: abnormal liver function test and prolonged prothrombin time), renal failure (glomerular filtration rate decrease ≥75%), heart diseases (i.e., coronary artery disease and congestive heart disease), history of convulsion, history of psychologic disorder or taking monoamine oxidase inhibitors, inability to understand the verbal numerical rating scale (VNRS) (VNRS: 0 = no pain, 10 = the most severe pain imaginable), chronic pain, or known allergy to drugs in our protocol.

The patients were randomized into 2 groups using block-of-four computer-generated randomization. They blindly received either parecoxib and nefopam (nefopam group) or parecoxib and normal saline (NSS) (control group). In the operating room, the patient underwent general anesthesia and standardized monitoring of noninvasive blood pressure, heart rate, pulse oximetry, and electrocardiography. The baseline pain score and all vital signs were obtained. After preoxygenation with 100% oxygen for 3–5 minutes, anesthesia was induced with propofol (1.5–2.5 mg/kg), atracurium (0.5 mg/kg) or cisatracurium (0.2 mg/kg), and fentanyl (1–2 mcg/kg). Subsequently, anesthesia was maintained with oxygen, nitrous oxide, seveflurane, or desflurane (1.0–1.5 minimum alveolar concentration (MAC)) with additional atracurium or cisatracurium as needed.

Nefopam 20 mg in NSS 100 mL and parecoxib 40 mg were administered intravenously to the participants in the nefopam group, while the control group received parecoxib 40 mg and NSS intravenously during closing the abdominal wall. Nefopam 20 mg in NSS 100 mL was injected intravenously every 6 hours (*h*) for 24 h postoperatively to the participants in the nefopam group, whereas the ones in the control group received NSS 100 mL every 6 h. Neostigmine (0.05 mg/kg) and atropine (0.02 mg/kg) were administered for reversal of neuromuscular relaxation before finishing the operation.

Pain intensity was assessed in the postanesthetic care unit using the VNRS at 1 h after operation. If the VNRS was more than 4 points, patients were administered morphine 3 mg until pain intensity less was than 4 points. Intravenous patient-controlled analgesia (IV-PCA) with morphine (protocol: morphine 1 mg/dose, lock-out interval of 5 minute, 4-hours limit of 40 mg and no basal infusion) was given for breakthrough pain within 24 h. The participants were then evaluated for postoperative pain using the VNRS at 6, 12, and 24 h after surgery by the nurses blinded to group assignment. Postoperative hemodynamic parameters such as blood pressure, heart rate, and adverse effects including nausea, vomiting, dry mouth, sweating, arrhythmia, and apnea were carefully evaluated and recorded.

Primary and secondary outcomes were dosages of morphine consumption within 24 h postoperative and the VNRS at 1, 6, 12, and 24 h after operation, as well as adverse effects, respectively.

The sample size was calculated based on our pilot study. The results showed that nefopam combined with parecoxib and parecoxib only reduced the 24 h postoperative morphine consumption from 15.0 ± 3.8 to 12.0 ± 4.7 mg. The number of 32 participants per group will provide 80% power to detect the equal difference in 24 h morphine consumption at a 2-sided alpha of 0.05. The definite number of participants per group was 36 after compensation of data loss.

All analyses were performed by SPSS for Windows Version 20.0. The data were expressed as mean, median, standard deviation (SD), and percentage as appropriate characteristics. Between-group comparisons were analyzed using the independent samples *t*-test or Mann–Whitney *U* test where appropriate. Categorical variables were compared between groups using the chi-square test. Pain scores were analyzed by mixed-effects maximum likelihood regression. A *P* value <0.05 was considered statistically significant.

## 3. Results

**Figure 1:**
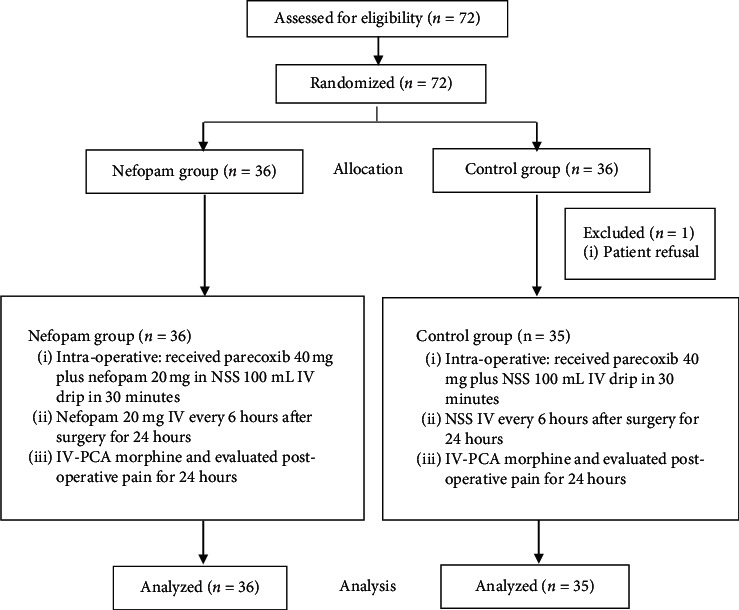
A study flow chart.

**Figure 2:**
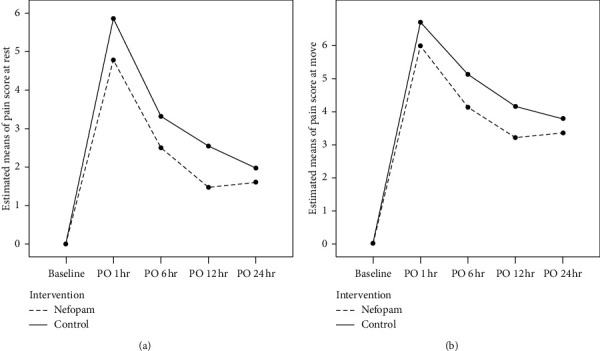
Estimated means of pain score analyzed by mixed-effects regression between nefopam and control at various time points. (a) At rest: no effect of intervention on the estimated mean pain score, *P* value = 0.058; (b) at movement: intervention, nefopam and control, affecting the estimated pain score, *P* value = 0.046.

Our study recruited 72 participants excluding 1 participant due to patient refusal (Figure 1). The demographic data, for example, ASA physical status and operative data, were not significantly different between both groups (Tables 1 and 2). There were no significant differences in the dosage of morphine consumption in 24-hour, side effects, and hemodynamic parameters between both groups (Table 3 and 4). Pain scores were not significantly influenced by the intervention, nefopam vs. control, as shown by mixed-effect maximum likelihood regression (*P*=0.058) (Figure 2.). Interestingly, the postoperative pain score on movement at 6 h and the pain score both at rest and on movement at 12 h were significantly lower in the nefopam group than the control groups (*P*=0.04, 0.03, and 0.03, respectively) (Table 5). Multiple comparisons of pain scores demonstrated that pain scores at 12 and 24 h after operation both at rest and movement were not significantly different which was similar between the nefopam and control group (Table 6).

## 4. Discussion

Our RCT study demonstrated the analgesic efficacy of multimodal analgesia using nefopam and parecoxib with IV-PCA over parecoxib with IV-PCA. It produced an additional efficacy by decreasing the postoperative pain score on movement at 6 h and the pain score both at rest and on movement at 12 h more than parecoxib only. However, no difference of morphine was found.

Multimodal analgesia has been developed and widely used postoperatively for many type of surgeries. Drugs used in this strategy include paracetamol, NSAIDs, opioid, and a newly agent nefopam. The aims of using multimodal analgesia are to decrease postoperative pain and undesirable effects of opioids [4]. Opioids acting centrally are widely used postoperatively despite of their adverse effects such as nausea, vomiting, bowel ileus, respiratory depression, and delirium. NSAIDs act at both the central and the peripheral level, by reducing prostaglandin production [12, 13]. Nefopam is a nonopioid analgesic drug with a centrally acting mechanism which has an equivalent analgesic effect to NSAIDs [1, 2, 6]. Yoon et al. [14] demonstrated the comparable analgesic efficacy using nefopam only and a combination of IV-PCA using of morphine and ketorolac in laparoscopic gynecologic surgery. Many studies showed lower incidence of postoperative nausea and vomiting in the nefopam group [11, 15, 16]. Several recent studies demonstrated the beneficial effect of nefopam, a significant postoperative opioid sparing effect, as an agent in multimodal analgesia [6, 8, 17, 18]. Jin el al. [19] reported significantly lower PCA fentanyl consumption and pain score in the nefopam-combined fentanyl group than the fentanyl group in open laparotomy. Mimoz et al. [20] performed an RCT study comparing control, nefopam, and propacetamol, all combined with morphine IV-PCA in hepatic resection. They reported that the postoperative pain intensity at 4 and 24 h and morphine consumption at immediately after extubation, 1–4, 4, and 24 h were significantly lower in the nefopam group. This finding is in line with the laparoscopic cholecystectomy study in which analgesic efficacies of ketorolac and nefopam combined with fentanyl were similar [21]. However, studies about multimodal analgesia using more than one NOA have been published scarcely.

Our RCT demonstrated the minimal enhancement of the analgesia of the combination of nefopam, parecoxib, and morphine IV-PCA for postoperative open major gynecologic surgery. The combined drugs significantly decrease pain intensity on movement at 6 h and both at rest and on movement at 12 h compared with parecoxib and morphine IV-PCA, but there was no significant difference of morphine consumption. Our results were inconsistent with the OCTOPUS study, a multicenter, double-blinded RCT, which compared different NOA combinations with morphine for postoperative analgesia [22]. The treatment groups in the OCTOPUS study included control, paracetamol (P), nefopam (N), ketorolac (K), PN, PK, and PNK. There was no significant difference of pain score and morphine consumption at 24 and 48 h after operation compared between NK vs. K. However, there were significant differences of (1) morphine consumption at 24 and 48 h between control vs. NPK and N vs. NPK and (2) the pain score at 24 h between control vs. NPK, N vs. NPK, and P vs. NPK. The study was stopped early because of the ethical concern of the investigators. The number of recruited participants was several fold lower than the calculated samples (*n*, 27 vs. 125 per group). Therefore, the analgesic effect when comparing between NSAID and nefopam could not be analyzed due to the inadequate power of the sample size. Moreover, many kinds of operation were evaluated which was different from our study.

The morphine consumption at 24 and 48 h between nefopam plus parecoxib and parecoxib only was not different but not the pain score on movement at 6 h and the pain score both at rest and on movement at 12 h. It could be possible because the pain score was not high, and then, the patients ignored administering morphine IV-PCA during changing the position. Therefore, a reduction in morphine consumption is not a good indicator of the benefit of adding analgesic. Moreover, data from a systemic study showed that morphine sparing was not changing parallel to morphine-related adverse effects [5]. Interestingly, a few prior studies evaluated the pain score at 6 or 12 h after operation as our study, a period at which patients often have severe pain.

Previous studies have reported sweating, tachycardia, and hypertension as common side effects of nefopam [6], but the incidence of these side effects in this study was similar in both groups. The frequency of adverse effects was not found to reduce by the combination of these, likely because of the relatively low incidence of adverse effects arising from this medications However, it is an advantage to use the minimal dose of each drug.

Limitations of the study were the subjective data of pain scores. Pain evaluation was a self-assessment using the VNRS that was individual dependent and multifactorial involvement such as pain evaluation at the bed time of the patients. Moreover, when the pain scores were low, it could not reflect the morphine consumption. Patient satisfaction should be evaluated in the future study for better analysis. Since we gave only 1 time of parecoxib which can maintain analgesia for 12 h, the latter 12 h postoperative analgesia could be explained by the effect of parecoxib and morphine IV-PCA. The future study may focus on other analgesic such as paracetamol, other kinds of NSAIDs, or even more multiple combined drugs.

## 5. Conclusions

Administration of nefopam combined with parecoxib is slightly better than parecoxib only for acute postoperative pain control at 6 and 12 h for gynecologic operations. This could help patients to ease their early ambulation. Morphine consumption in 24 hours was not significantly different in both groups with comparable adverse effects.

## Figures and Tables

**Table 1:** Demographic data.

	Nefopam (*N* = 36)	Control (*N* = 35)	*P* value
Age (years)	41.9 ± 9.6	42.9 ± 8.1	0.667
Weight (kg)	59.8 ± 10.7	61.9 ± 10.2	0.412
Height (m)	1.6 ± 0.1	1.6 ± 0.1	0.226
BMI (kg.m^−2^)	24.5 ± 4.4	24.8 ± 3.9	0.777

ASA			
I	13 (36.1%)	12 (34.3%)	0.872
II	23 (63.9%)	23 (65.7%)	

Diagnosis			
Adenomyosis	3 (8.3%)	11 (31.4%)	0.014
Cervix cancer	1 (2.8%)	0 (0%)	1.000
Endometrial cyst	2 (5.6%)	0 (0%)	0.493
Endometrium cancer	1 (2.8%)	0 (0%)	1.000
Endometrium hyperplasia	0 (0%)	1 (2.9%)	0.493
Myoma uteri	24 (66.7%)	18 (51.4%)	0.192
Ovarian mass	5 (13.9%)	5 (14.3%)	1.000

Type of operation			
TAH	36 (100.0%)	35 (100.0%)	0.117
TAH with BSO	26 (72.2%)	19 (54.3%)	—
Operation time (min)	164.2 ± 53.8	162.3 ± 62.9	0.893
Blood loss (mL)	344.4 ± 383.9	437.7 ± 508.7	0.385

Type of wound incision			
Midline	12 (33.3%)	8 (22.9%)	0.327
Pfannenstiel	24 (66.7%)	27 (77.1%)	
Incision length (cm)	11.9 ± 2.9	11.8 ± 2.8	0.898

Data are represented as mean ± standard deviation or number (%) where appropriate. Abbreviations: TAH, total abdominal hysterectomy; BSO, bilateral salpingo-oophorectomy.

**Table 2:** Intraoperative characteristics.

	Nefopam (*N* = 36)	Control (*N* = 35)	*P* value
Type of operation			
TAH only	10 (27.8%)	16 (45.7%)	0.117
TAH with BSO	26 (72.2%)	19 (54.3%)	—
Operation time (min)	164.2 ± 53.8	162.3 ± 62.9	0.893
Intraoperative fentanyl use (mg)	86.9 ± 27.1	85.1 ± 30.7	0.910
Blood loss (mL)	344.4 ± 383.9	437.7 ± 508.7	0.385

Type of wound incision			
Midline	12 (33.3%)	8 (22.9%)	0.327
Pfannenstiel	24 (66.7%)	27 (77.1%)	
Incision length (cm)	11.9 ± 2.9	11.8 ± 2.8	0.898

Data are represented as mean ± standard deviation or number (%) where appropriate. Abbreviations: TAH, total abdominal hysterectomy; BSO, bilateral salpingo-oophorectomy.

**Table 3:** Morphine consumption of nefopam and control groups.

	Nefopam (*N* = 36)		Control (*N* = 35)		Mean difference	*P* value
	Mean	SD	Mean	SD	(95% CI)	
Morphine consumption in 24 hours (mg)	19.58	9.64	19.49	10.05	1.00 (−4.56, 4.76)	0.97

Abbreviations: PO, postoperation; SD, standard deviation; CI, confidence interval.

**Table 4:** Adverse effects of nefopam and control groups.

	Nefopam (*N* = 36)	Control (*N* = 35)	*P* value
Nausea and vomiting	14 (38.9%)	12 (34.3%)	0.687
Dry mouth	6 (16.7%)	4 (11.4%)	0.735
Sweating	3 (8.3)	0 (0.0%)	0.239
Arrhythmia	1 (2.8%)	0 (0.0%)	1.000

Data are represented as number (%).

**Table 5:** Pain score assessment during the operation and after the operation.

		Nefopam (*N* = 36)		Control (*N* = 35)		Mean difference	*P* value
		Mean	SD	Mean	SD	(95% CI)	
Baseline	At rest	0	0	0	0	0(0, 0)	1.00
	On movement	0	0	0.03	0.17	−0.03 (−0.09, 0.03)	0.32

PO 1 hour	At rest	4.78	3.08	5.86	3.29	−1.08 (−2.59, 0.43)	0.16
	On movement	6.00	3.10	6.71	2.94	−0.71 (−2.14, 0.72)	0.32

PO 6 hours	At rest	2.50	2.15	3.31	2.42	−0.81 (−1.90, 0.27)	0.14
	On movement	4.14	2.11	5.14	1.80	−1.00 (−1.94, −0.07)	0.04

PO 12 hours	At rest	1.47	1.80	2.54	2.15	−1.07 (−2.00, −0.13)	0.03
	On movement	3.22	1.84	4.17	1.74	−0.95 (−1.80, −0.10)	0.03

PO 24 hours	At rest	1.61	1.67	1.97	2.11	−0.36 (−1.26, 0.54)	0.43
	On movement	3.36	1.69	3.80	1.78	−0.44 (−1.26, 0.38)	0.29

PO, postoperation; SD, standard deviation; CI, confidence interval.

**Table 6:** Multiple comparison of pain scores during at rest and movement between nefopam and control groups.

Parameter		Diff	95% CI	*P* value	Parameter		Diff	95% CI	*P* value
At rest					At rest				
Nefopam					Control				
Baseline	PO 1 hr	−4.8	−6.3, −3.2	<0.001	Baseline	PO 1 hr	−5.9	−7.5, −4.2	<0.001
	PO 6 hrs	−2.5	−3.5, −1.4	<0.001		PO 6 hrs	−3.3	−4.5, −2.1	<0.001
	PO 12 hrs	−1.5	−2.4, −0.5	<0.001		PO 12 hrs	−2.5	−3.6, −1.4	<0.001
	PO 24 hrs	−1.6	−2.4, −0.8	<0.001		PO 24 hrs	−1.9	−3.0, −0.9	<0.001
PO 1 hr	PO 6 hrs	2.3	0.6, 3.9	0.002	PO 1 hr	PO 6 hrs	2.5	1.1, 4.0	<0.001
	PO 12 hrs	3.3	1.6, 5.0	<0.001		PO 12 hrs	3.3	1.7, 4.9	<0.001
	PO 24 hrs	3.2	1.6, 5.7	<0.001		PO 24 hrs	3.9	2.4, 5.4	<0.001
PO 6 hrs	PO 12 hrs	1.0	0.2, 1.8	0.004	PO 6 hrs	PO 12 hrs	0.8	−0.3, 1.8	0.312
	PO 24 hrs	0.9	0, 1.8	0.062		PO 24 hrs	1.3	0.4, 2.3	0.002
PO 12 hrs	PO 24 hrs	0.1	−0.5, 0.8	1.000	PO 12 hrs	PO 24 hrs	0.6	−0.3, 1.4	0.576
									

At movement					At movement				
Nefopam					Control				
Baseline	PO 1 hr	−6.0	−7.5, −4.4	<0.001	Baseline	PO 1 hr	−6.7	−8.2, −5.2	<0.001
	PO 6 hrs	−4.1	−5.2, −3.1	<0.001		PO 6 hrs	−5.1	−6.0, −4.2	<0.001
	PO 12 hrs	−3.2	−4.1, −2.3	<0.001		PO 12 hrs	−4.1	−5.0, −3.3	<0.001
	PO 24 hrs	−3.4	−4.2, −2.5	<0.001		PO 24 hrs	−3.8	−4.7, -2.9	<0.001
PO 1 hr	PO 6 hrs	1.9	0.3, 3.4	0.010	PO 1 hr	PO 6 hrs	1.6	0.3, 2.9	0.009
	PO 12 hrs	2.8	1.1, 4.4	<0.001		PO 12 hrs	2.5	0.9, 4.2	<0.001
	PO 24 hrs	2.6	1.4, 3.9	<0.001		PO 24 hrs	2.9	1.4, 4.4	<0.001
PO 6 hrs	PO 12 hrs	0.9	0.08, 1.8	0.024	PO 6 hrs	PO 12 hrs	1.0	0, 1.9	0.034
	PO 24 hrs	0.8	−0.1, 1.7	0.157		PO 24 hrs	1.3	0.4, 2.3	0.001
PO 12 hrs	PO 24 hrs	−0.1	−1.0–0.7	1.000	PO 12 hrs	PO 24 hrs	0.4	−0.2, 0.9	0.567

PO, postoperation; SD, standard deviation; CI, confidence interval; Diff, difference; hr, hour.

## Data Availability

All data generated or analyzed during this study are included in this manuscript.
